# Comparison of surgical outcomes and prognosis between wedge resection and simple Segmentectomy for GGO diameter between 2 cm and 3 cm in non-small cell lung cancer: a multicenter and propensity score matching analysis

**DOI:** 10.1186/s12885-021-09129-0

**Published:** 2022-01-16

**Authors:** Yaodong Zhou, Tao Yu, Yixin Zhang, Liqiang Qian, Qing Xia

**Affiliations:** 1 Yaodong Zhou, Department of Thoracic Surgery, Fudan University Shanghai Cancer Center, 270 Dong-An Road, Shanghai, 200032 People’s Republic of China; 2 Department of Plastic and Reconstructive Surgery, Shanghai Ninth People’s Hospital affiliated to Shanghai Jiaotong University, Shanghai, 200011 China; 3 Department of Thoracic Surgery, Shanghai Chest Hospital, Shanghai, 200030 China; 4 Department of Neonate, Children’s Hospital of Fudan University, Shanghai, 201102 China

**Keywords:** NSCLC, Segmentectomy, Wedge resection, Ground-glass opacity, Propensity score matching

## Abstract

**Background:**

As segmentectomy had become commonly used for Non-Small Cell Lung Cancer (NSCLC) treatment, which had the advantages of radical operation, however, it remains controversial owing to procedural complexity and risk of increased complications compared with wedge resection. We evaluated operative and postoperative outcomes of simple segmentectomy compared to wedge resection in ground-glass opacity (GGO) diameter between 2 cm and 3 cm NSCLC.

**Methods:**

We retrospectively reviewed 1600 clinical GGO diameter between 2 cm and 3 cm NSCLC patients who received simple segmentectomy and wedge resection between Jan 2011 and Jan 2015. Participants were matched 1:1 on their propensity score for two groups. Clinic-pathologic, operative, and postoperative results of two groups were compared.

**Results:**

After using propensity score methods to create a matched cohort of participants with simple segmentectomy group similar to that wedge resection, there were no significant differences detected in tumor size, margin distance, histology, age, sex, preoperative comorbidities and preoperative pulmonary function. Overall complications in simple segmentectomy group were more than wedge resection group (21% vs 3%, *p* = 0.03). Median operative time (110.6 vs. 71.2 min; *p* = 0.01) and prolonged air leakage (12% vs. 3%; *p* = 0.02) was significantly longer in the simple segmentectomy group. There was no difference in recurrence free survival (RFS) and overall survival (OS) of 5-years between simple segmentectomy group and wedge resection group. Postoperative pulmonary function in simple segmentectomy group recovered more slowly than wedge resection group.

**Conclusion:**

Wedge resection may have comparable efficacy as simple segmentectomy for GGO diameter between 2 cm and 3 cm NSCLC, but lead to less complications, less surgical procedure and faster recovery of pulmonary function.

**Supplementary Information:**

The online version contains supplementary material available at 10.1186/s12885-021-09129-0.

## Background

Over the last few decades, pulmonary lobectomy with systemic meditational lymphadenectomy has been chosen as the standard surgical therapy for NSCLC, which found that sublobar resection was associated with inferior overall survival and high recurrence chance compared to pulmonary lobectomy [1]. After that, sublobar resection is just performed only for patients unable to tolerate lobectomy. However, in recent ten years, the widespread of the lung cancer CT-screening programs together with progression in imaging, have lead to a large number of GGO diagnosed [2]. Many researches have reported that sublobar resection for small size tumors of stage I NSCLC can yield similar outcomes to those patients undergoing lobectomy [3, 4]. Therefore, pulmonary segmentectomy probably could become the standard treatment choice for NSCLC with early stage recently, which is selected more frequently [5]. However, wedge resection, still as one of sublobar resection type, has been criticized for its marginal recurrence and short of thoroughness without lymph node dissection, which affects its clinical value [4].

Segmentectomy could be further subdivided into simple segmentectomy and complex segmentectomy, according to surgical procedure. Complex segmentectomy needed to create artificial intersegmental planes, with more complex procedures, such as resection of the Right Superior (RS) (1 + 3), RS3, Left Inferior (LI) 9, and LI (9 + 10) segments, etc. Simple segmentectomy, usually called “classic segmentectomy”, easy to create linear intersegmental plane, was more regular in operation, such as RS 2, Right Inferior(RL)6, RL(7 + 8 + 9 + 10), LS(1 + 2 + 3) segments, left lingular segment, LI 6 and LI(7 + 8 + 9 + 10). Though simple segmentectomy do not need complex procedure, surgeons still often isolate and divide suitable segmental vein, artery, bronchus, and in some cases separate into lung parenchyma especially suffering the poor intersegmental plane. We focus on simple segements because they are the classic and most widely used surgical procedures for segements. However, higher complications rates, such as prolonged air leakage, longer stay in hospital, and length of chest tube drainage are concerned [6]. Wedge-shaped resection of the lung has the advantages of simpler procedure, less operation time and controllable margin. Especially with preoperative CT-guiding puncture location, it will become easier.

The debate often centers on the surgical procedure required for ground-glass nodules 1 to 2 cm in size. Few studies have explored the clinical outcomes of simple segmentectomy vs wedge resection in GGO diameter between 2 cm and 3 cm NSCLC. We evaluated pre-operative condition, operative, postoperative outcomes, prognosis and variations of pulmonary function in those patients who underwent simple segmentectomy versus wedge resection.

## Patients and methods

### Study population

This retrospective study comprised of patients(*n* = 1600) with clinical GGO diameter between 2 cm and 3 cm NSCLC who underwent lung operation at the Shanghai Ninth People’s Hospital Affiliated to Shanghai Jiaotong University, Shanghai Chest Hospital and Children’s hospital of Fudan University between January 2011 and January 2015. The present study was approved by the Research Ethics Committee of Shanghai Jiaotong University (Shanghai, People’s Republic of China), and written informed operation-consent was obtained from all patients. Patient inclusion criteria were acceptable to tolerate pulmonary lobectomy as evaluated by cardiorespiratory fitness tests. All the operations were purely simple segmentectomy or wedge resection. We excluded patients in whom the surgeon mixed the operation, such as lobectomy + wedge resection or segmentectomy + wedge resection.

### Propensity score methods

Propensity score matching was used to reduce these selection biases when estimating the association of different sublobar resection with complications and prognosis. To calculate the propensity score, we used logistic regression to obtain the predicted probability of exposure (i.e. tumor size, margin distance, tumor site, surgical types). The propensity scores were obtained based on the most accurate and clinically relevant model including the baseline variables: age, sex, preoperative cardiopulmonary function, smoking status, surgical types, histology, and tumor site. Using this model, we could create propensity score weights for all participants to guarantee there were no missing values.

Patients with simple segmentectomy group were matched 1-to-1 without replacement using a varying-width caliper-matching algorithm (5-to-1 digit matching). The propensity scores were then checked to ensure they were balanced across the simple segmentectomy and wedge resection groups. The balance in covariates was assessed before and after matching using standardized differences. Importantly, standardized differences of 0.2, 0.5 and 0.8 were deemed to represent tiny, medium and huge differences, respectively [7].

### Preoperative evaluation

All the patients received careful preoperative staging with CT, cardio-pulmonary function testing within 4 weeks of the surgical procedure. The emission computed tomography (ECT), Positron Emission Tomography-Computed Tomography (PET-CT), brain magnetic resonance imaging and tracheabronchoscopy was dependent by the surgeon who judged it whether or not necessary. Tumor stage was determined according to the size of tumor, nodes, and metastasis classification of malignant tumors on the basis of 8th edition [8]. All the diameter of ground grass opacity nodule (GGO) or solid nodules was between 2 and 3 cm. Patients with solid component size 2 cm or over were excluded.

### Surgical procedure

In general, the choice between wedge resection and segmental resection is a decision made by each surgeon based on expert consensus or guidelines combined with his own judgment. Surgical Procedure mainly involved anatomic pulmonary segmentectomy or resection of wedge to guarantee acceptable resection margin, and to isolate hilar, segmental, and excise or sample mediastinal lymph nodes to confirm N0 treatment. All the patients in the wedge resection group mostly need to receive CT-guided needle localization. All the operations were performed by VATS with two incisions or three incisions. Surgeons were allowed to mark and divide the intersegmental plane, using argon beam, electrocautery, or segmental stapling to secure adequate surgical margin and the inflation–deflation line. After resection, the surgeon should confirm the result of pathology and surgical margin again. If the surgical margin was not satisfied, additional wedge resection was required to extend the distance.

### Follow-up

Perioperative condition data and intra-operative data were collected from the hospital medical records, anesthesia, and operating room records for each patient. Postoperative complications were summarized using the Clavien–Dindo classification [9]. Relapse patterns mainly include local relapse, regional relapse and distant relapse. Local relapse mainly refers to margin relapse, region relapse mainly refers to local lymph node relapse, both of which are the focus of our follow-up. All the patients underwent pulmonary function test from pre-operation to postoperative at 3, 6 and 12 months.

### Statistical analysis

The collected data are presented as numbers or median (interquartile range) or mean value. The statistical method used for comparing RFS and OS is log-rank. Differences in the multifarious variables between simple segmentectomy and wedge resection groups were assessed by Fisher’s test or the Mann-Whitney U test, in which measurement data were used by Paired t-test and count data were used by McNemar’s test. Prognostic factors for survival were identified using a multivariate Cox proportional hazards model. Pulmonary function changes after simple segmentectomy or wedge section group were compared by repeated-measures analysis of variance and time-dependent variations in the forced vital capacity (FVC), forced expiratory volume in 1 s (FEV1.0), predicted diffusing capacity of the lung for carbon monoxide (DLCO%) and peak expiratory flow (PEF) were evaluated. All tests were two-sided and a *p* value less than or equal to 0.05 was set as statistical significance. All the data was analyzed statistically using SPSS 19.0 (IBM, Armonk, NY, USA).

## Results

**Fig. 1:**
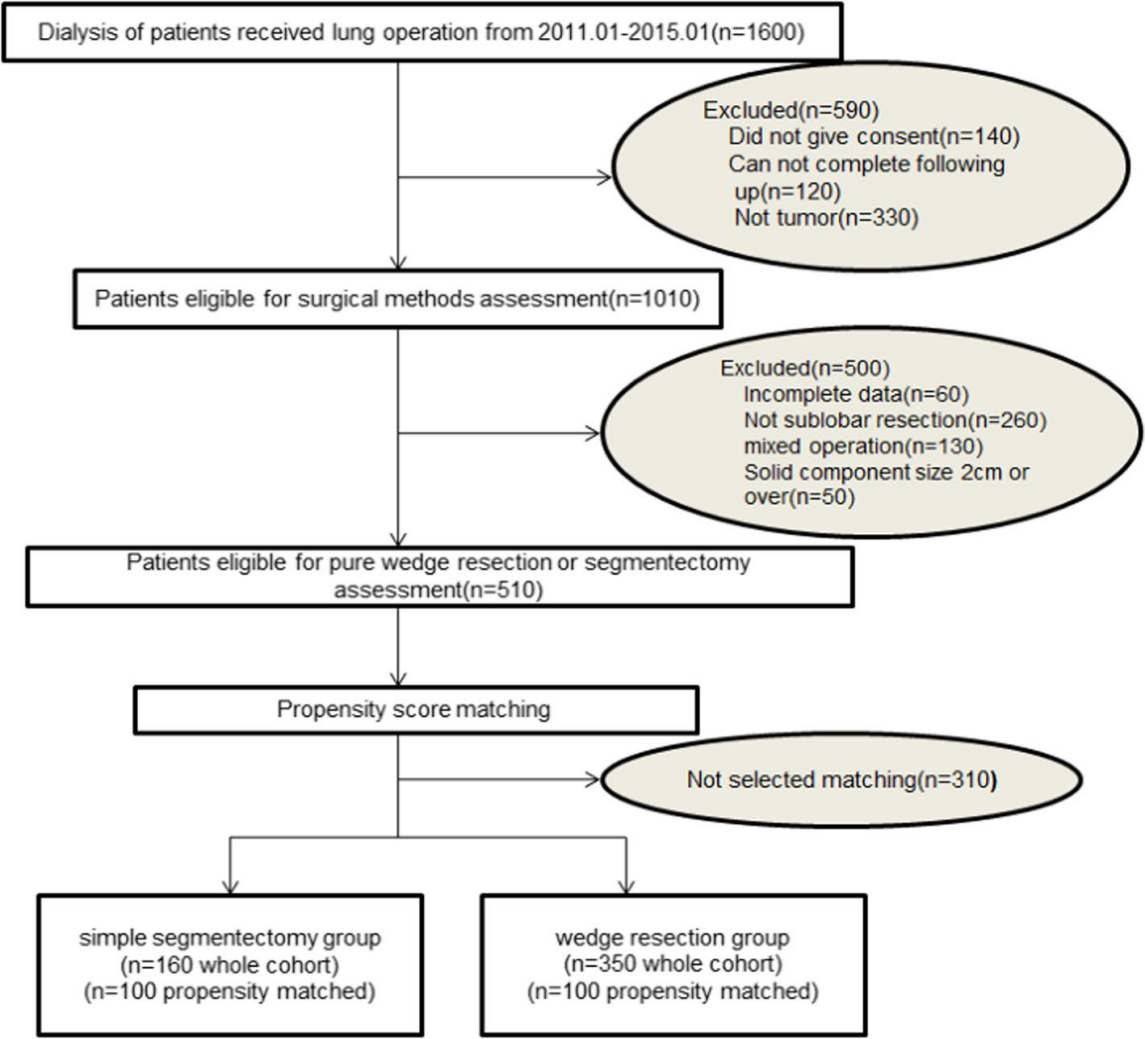
Flow chart of study enrolment and exclusion

Overall, 1600 patients who had undergone lung surgery were assessed for eligibility (Fig. 1). After excluding 140 who did not provide consent, 120 due to incomplete following up, and 330 benign tumors, our study involved 1010 participants. Then, we excluded 60 because of incomplete clinical data, 310 who do not receive sublobar resection and 130 mixed different operations. There were 510 patients left for propensity score matching (Supplementary table), which resulted in simple segmentectomy group (*n* = 100) versus the wedge resection group (n = 100) forming 1:1 matching (Fig. 1, Table 1). Significantly different with full cohort, the matching reduce the selection bias and make the variables similar, such as age, gender, tumor size, margin distance, smoking status, tumor site, histology, preoperative cardiopulmonary function and so on (Table 1).

**Table 1:** Baseline characteristics of study participants according to different operation group status in unmatched and matched cohorts

Variables	Full cohort			After propensity score matching		
	Simple segmentectomy group (*n* = 160)	Wedge resection group (*n* = 350)	Standardized Difference^a^	Simple segmentectomy group (*n* = 100)	Wedge resection group (*n* = 100)	Standardized difference^a^
General data						
Age	55.4(13.5)	62.9(16.2)	0.28	57.2(14.1)	57.7(14.2)	0.002
Male	95(59.3%)	192(54.8%)	0.91	58(58%)	53(53%)	0.015
Tumor size(cm)	2.92(0.2)	2.64(0.17)	0.48	2.73(0.19)	2.71(0.19)	0.011
Solid component size	1.54(0.14)	1.92(0.19)	0.37	1.78(0.17)	1.82(0.18)	0.013
Smoking status						
Ever	77(48.1%)	198(56.5%)	−0.39	47(47%)	49(49%)	0.003
Margin(mm)	13.2(4.2)	25.7(5.6)	−0.66	19.5(3.6)	22.4(4.1)	0.002
Median operative time(min)	121.7(25.3)	62.9(14.3)	0.53	110.6(19.5)	71.2(17.9)	0.013
Blood loss (ml)	210(45.1)	120(21.7)	0.77	150(26.8)	130(24.2)	0.001
Dissected lymph nodes(n)	6(0.57)	2.5(0.29)	0.55	4(0.34)	1.5(0.31)	0.006
Tumor Site						
RUL	33(20.6%)	81(23.1%)	0.456	21(21%)	19(19%)	0.01
RML	0	14(4%)	0.65	0	3(3%)	0.018
RLL	31(19.3%)	97(27.7%)	−0.541	23(23%)	26(26%)	−0.009
LUL	48(30%)	69(19.7%)	−0.42	37(37%)	33(33%)	0.01
LLL	48(30%)	89(25.4%)	−0.47	19(19%)	19(19%)	0
Histology						
Adenocarcinoma	142(88.7%)	287(82%)	−0.41	91(91%)	89(89%)	0.01
AIS	46(28.8%)	98(28%)	−0.33	29(29%)	26(26%)	0.05
MIA	57(35.6%)	123(35.1%)	−0.51	32(32%)	34(34%)	0.04
IA	39(24.3%)	66(18.9%)	0.21	30(30%)	29(29%)	−0.012
Pulmonary function (%)	58.6%(4.9)	53.7%(3.7)	0.35	57.3%(4.5)	54.2%(3.8)	0.05
Poor pulmonary function^b^	34(21.2%)	81(23.1%)	−0.44	21(21%)	24(24%)	0.02
Ejection fraction (%)	67.1%(5.8)	60.2%(5.1)	0.38	64.6%(5.6)	61.7%(5.1)	0.041
Poor ejection fraction^c^	22(13.8%)	31(8.9%)	0.31	16(16%)	13(13%)	0.023

Data are expressed as mean (SD) or number (%). ^a^ Standardized differences of 0.2, 0.5 and 0.8 were deemed to represent tiny, medium and huge differences, respectively. These differences do not denote statistical significance

^b^ Poor pulmonary function was defined as an FEV1/FVC ratio of< 50%

^c^ Poor ejection fraction was defined as a left ventricular ejection fraction of< 50%

### Clinicopathologic data in patients

In the two groups, adenocarcinoma accounts for the highest percentage of pathology (total *n* = 180, 90%), including adenocarcinoma in situ (AIS), microinvasive adenocarcinoma (MIA), and invasive adenocarcinoma (IA), followed by squamous cell carcinoma (total *n* = 13, 6.5%, Table 2). There were no significant differences in clinic-pathologic data, including age, sex, number of solid nodules, mixed GGO (mGGO), pure GGO (pGGO), preoperative pulmonary function testing, pathology, or tumor size between two groups. Varieties of comorbidities, such as coronary artery disease, chronic obstructive pulmonary disease (COPD) and diabetes mellitus were similar in both groups (Table 2).

**Table 2:** Patient and tumor characteristics of simple segmentectomy and wedge resection

Variables	simple segmentectomy *n* = 100	wedge resection *n* = 100	*p* value
Age (years)			0.14
> 60	65	62	
≤ 60	35	38	
Sex			0.22
Male	58	53	
Female	42	47	
Comorbidities			0.08
Coronary artery disease	13	15	
Diabetes mellitus	17	20	
COPD	17	14	
Tumor size (mm)	26.7	25.9	0.43
CTR	0.78	0.75	0.69
Clinical Stage			
cTisN0	25	24	0.39
cT1miN0	36	34	0.67
cT1aN0	16	17	0.54
cT1bN0	14	14	0.78
mGGO	45	43	0.54
pGGO	55	57	0.66
Pulmonary function ^a^	57.3%	54.2%	0.21
Ejection fraction	64.6%	61.7%	0.15
Histology			
Adenocarcinoma	91(91%)	89(89%)	0.09
AIS	29	26	
MIA	32	34	
IA	30	29	
Squamous cell carcinoma	6(6%)	7(7%)	0.07
Others	3(3%)	4(4%)	0.33
Pathologic stage			
pTisN0	29	26	0.34
pT1miN0	32	34	0.43
pT1aN0	12	14	0.22
PT1bN0	18	15	0.26
Visceral pleural invasion	1	1	
Lymphovascular invasion	3	2	

^a^ FEV1/FVC ratio

*COPD* Chronic obstructive pulmonary disease, *GGO* Ground-glass opacity, *FEV1* Forced expiratory volume in 1 s, *FVC* Forced vital capacity, *CTR* Consolidation tumor ratio

### Surgical outcomes

In our study, simple segmentectomy included resection of the RS2, RS6, LS(1 + 2 + 3), LS6, or lingual segment (Table 3). Median operative time was obviously longer for the simple segmentectomy group versus the wedge resection group (110.6 min vs 71.2 min, *p* = 0.002, Table 4). However, estimated blood loss was not significantly different (150 ml vs. 130 mL, *p* = 0.07). Median surgical margins were similar (19.5 mm vs 22.4 mm, *p* = 0.7). No postoperative marginal recurrence occurred in either group. Mean number of dissected lymph nodes during simple segmentectomy group was more than wedge resection group (4.0 VS 1.5 *=* 0.003).

**Table 3:** Tumor locations of simple segmentectomy group and wedge resection group

Locations	simple segmentectomy n = 100	wedge resection n = 100
Right upper		19
S2	21	
S3	0	
Right middle	0	3
Right lower		26
S6	20	
S(7 + 8 + 9 + 10)	3	
Left upper		33
S(1 + 2 + 3)	25	
lingular segment	12	
Left lower		19
S6	17	
S(7 + 8 + 9 + 10)	2

**Table 4:** Operative and postoperative data of simple segmentectomy and wedge resection

Variables	simple segmentectomy n = 100	wedge resection *n* = 100	*p* value
Operative data			
time (min)	110.6	71.2	0.002
blood loss (ml)	150	130	0.07
margin (mm)	19.5	22.4	0.07
dissected lymph nodes (n)	4	1.5	0.003
staple^1^ (n)	4.2	4.7	
Postoperative data			
stay in hospital (day)	5.2	3.1	0.043
hospitalization expenses ($)	5020	3900	0.035
drainage (day)	3.4	2.2	0.04
air leakage (> 7 day)	12	3	0.02
Postoperative complications			
overall	21(21%)	3(3%)	0.031
pulmonary infection	7	1	
atelectasis	8	2	
chylothorax	5	0	
bleeding	1	0	
Relapse patterns			0.22
local relapse	0	0	
region relapse	4	3	
distant relapse	2	2	

^1^Numbers of staple for complete the operation

### Postoperative outcomes

In the postoperative outcome analysis, no deaths occurred in perioperative period after segmentectomy and wedge resection. The median hospital stay and median length of drainage in simple segmentectomy group was longer than wedge resection group (5.2 vs 3.1, *p* = 0.043; 3.4 vs 2.2, p = 0.043, respectively, Table 4). The average hospitalization expenses in simple segmentectomy group was more than wedge resection group (5020$ vs 3900$, *p* = 0.035). Overall complications occurred in the simple segmentectomy group were more than wedge resection group (21% vs 3%, *p* = 0.031, Table 4).

### Prognosis of 5-years

**Fig. 2:**
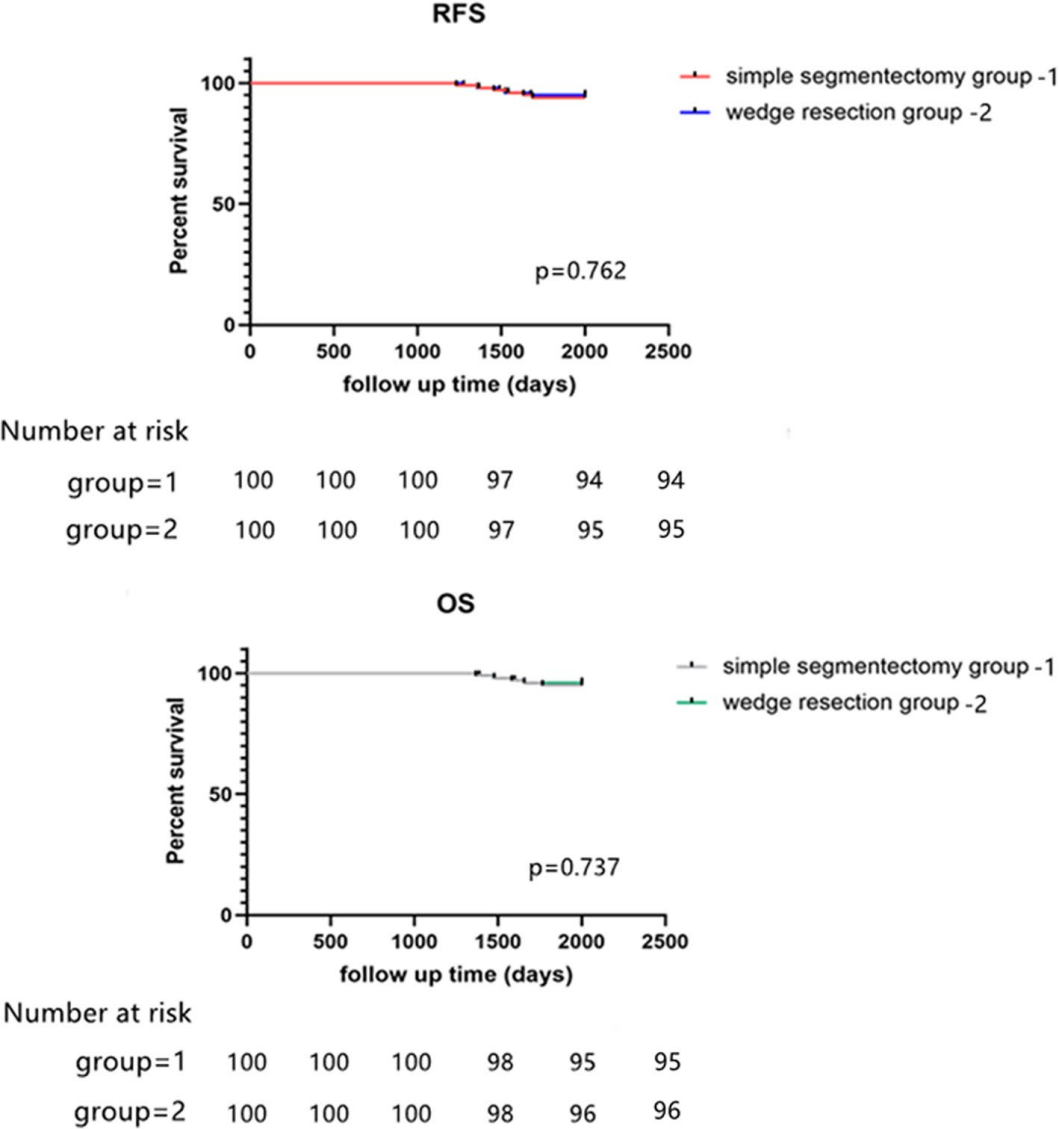
The 5-year RFS and OS in simple segmentectomy group was 94 and 95%, and in wedge resection group was 95 and 96%. There was no difference on the RFS and OS between simple segmentectomy group and wedge resection group

All the patients were followed up for 5 years after operation. The median follow-up time was 62 months. The time of reexamination of chest CT was 6, 18, 36, 48, 60 and 84 months after surgery, respectively. The 5-year RFS and OS in simple segmentectomy group was 93.1 and 91.9%, and in wedge resection group was 96 and 95.7% before propensity score matching (Supplementary figure). Importantly, after propensity score matching, in our research, the 5-year recurrence free survival (RFS) and overall survival (OS) in simple segmentectomy group was 94 and 95%, and in wedge resection group was 95 and 96% (Fig. 2). There was no difference on prognosis between two groups (*p* = 0.08). There was no margin relapse in the 5-years. Eleven patients respectively suffered intrapulmonary metastasis (2), mediastinal lymph node metastasis (7), pleural metastasis (1), and brain metastasis (1). Intrapulmonary metastasis occurred in wedge group and simple segmentectomy group respectively. There were 3 patients with mediastinal lymph node metastasis in the wedge group, 4 in the simple segmentectomy group, pleural metastasis in the wedge group, and brain metastasis in the simple segmentectomy group. Nine of the 11 patients died from tumor-related causes. There was no significant difference in the patterns of relapse between the two groups (*p* = 0.22, Table 4). Based on the univariate analysis, surgical method, complications, surgical margin, lymph nodes, were significant prognostic factors. A multivariate analysis identified surgical margin (relative risk 4.23, *p* = 0.003) and lymph nodes (relative risk 6.34, *p* = 0.001) as independent prognostic factors for OS.

### Postoperative changes of pulmonary functions

**Fig. 3:**
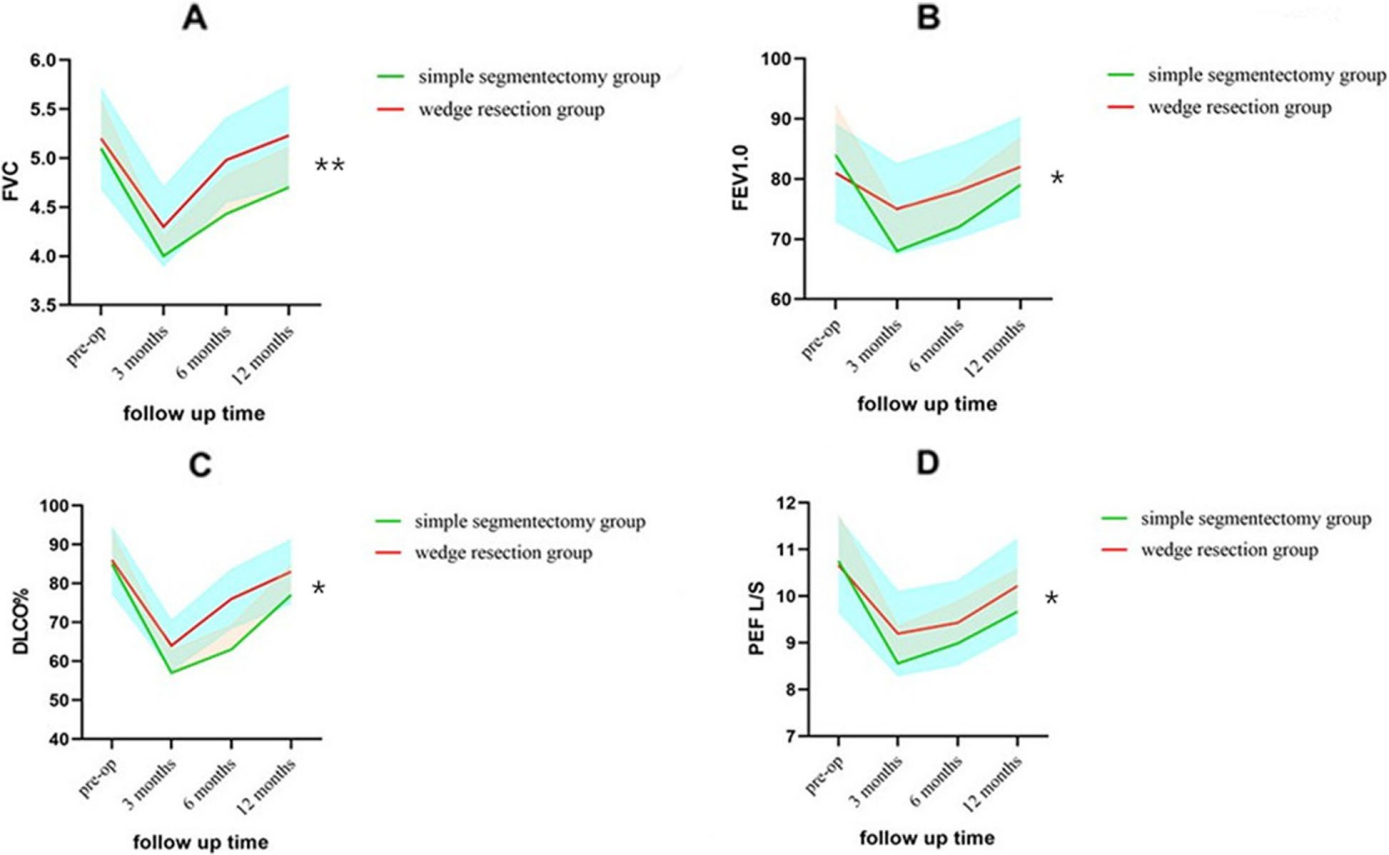
The pulmonary function changes in simple segmentectomy group and wedge resection group. **A**-**D** There were forced vital capacity (FVC), forced expiratory volume in 1 s (FEV1.0), predicted diffusing capacity of the lung of carbon monoxide percentage (%DLCO) and peak expiratory flow (PEF) preoperatively at 3, 6 and 12 months postoperatively in patients undergoing simple segmentectomy group and wedge resection group

Finally, we compared the pulmonary functional variations in the simple segmentectomy group and wedge resection (Fig. 3). All the 200 patients received the pulmonary functions test and there is no censoring. During the postoperative period, we observed both the two groups showed a different course of lung function in the test of FVC, FEV1.0, DLCO% and PEF after postoperative 3 months, 6 months and 12 months. However, patients in the wedge resection group showed up better recovery of pulmonary function compared with simple segmentectomy group (*p* = 0.04, *p* = 0.032, p = 0.03, 0.025, respectively; Fig. 3).

## Discussion

In recent decades, there has been so much research focusing on short and long term prognosis and complications between pulmonary lobectomy, segmentectomy and wedge resection [5, 10]. Controversies still exist on the most valuable choice. Mounts of literature in the world has proved the use of sublobar resection for patients with small, peripheral NSCLC [2, 11]. Okada et al. reported the segmentectomy patients reached almost the same 5-year survival rate (87.1% vs 87.7%), which is almost equivalent to lobectomy group in patients with T1N0 tumors [12]. In addition, overall recurrence rates were also similar after segmentectomy (17.6%) and lobectomy (16.7%) [4]. Therefore, sublobar resection had become a more conventional option for those early stage NSCLC patients.

Sublobar resection could be subdivided into segmentectomy and wedge resection. According to surgical process, segmentectomy included simple and complex segmentectomy. In our study, we just choose simple segmentectomy or we call it “classical segmentectomy”. Actually, simple segmentectomy is more conventional and common used in many centers. However, because of the worry about lymph node and margin relapse, wedge resection was taken to perform seriously and deliberately. Some studies reported that wedge resection suffered more incidences of local recurrence and lower OS than segmentectomy [13, 14]. It restricted the application of wedge resection. However, we explored the pre-operative, operative and postoperative outcomes of patients undergoing simple segmentectomy and wedge resection, including morbidity, complications, surgical margin, lymph nodes, prognosis and recovery of pulmonary function between two groups. No patient in both groups died during the perioperative period. Compared with simple segmentectomy group, wedge resection group entirely showed less operative time (71.2 min vs 110.6 min, *p* = 0.002), drainage time (2.2 vs 3.4 days, *p* = 0.04), stay in hospital (3.1 days vs 5.2 days, *p* = 0.043) and hospital expense (3900$ vs 5020$, *p* = 0.035). Moreover, there were similar 5-years OS (Fig. 2) in both groups. These complications are less and OS are higher than in previous reports after segmentectomy or wedge resection [15, 16]. The wedge resection group showed remarkably priority on simple segmentectomy group in our study. From our study, to stage GGO diameter between 1 cm and 2 cm, segmentectomy is overkill. A wedge with negative margins seems to be more than sufficient. These results highlight the favorable biology (and of course less aggressiveness) of this kind of tumor in the Asian population, which could not be reproducible for the rest of the world (Europe or America).

Several researches reported that affirming satisfactory surgical margins was strikingly significant to effectively prevent margin relapse, even though the resection margin has a negative pathological result [17]. It is still uncertain what the margin is enough in the operation to avoid relapse again. Generally speaking, 20 mm margin in an inflated lung and 15 mm in a deflated lung were believed to be safe and suitable [18]. In addition, one prospective and multicenter study has reported that margin distance greater than the tumor diameter was considered optimal for avoiding margin relapse [19]. In the study of Tsutani et al., postoperative recurrence occurred in 36 of 195 patients (18.5%) undergoing wedge resection and 14 of 262 patients (5.3%) undergoing segmentectomy. Cancer control was better in segmentectomy than in wedge resection [20]. Suzuki et al. reported that Median pathological surgical margin was 15 mm (0–55) and the 5-year relapse-free survival was 99.7% (90% confidence interval, 98.3–99.9). Sublobar resection with enough surgical margin offered sufficient local control and relapse-free survival for lung cancer clinically resectable N0 staged by computed tomography with 3 or fewer peripheral lesions 2.0 cm or less amenable to sublobar resection and with a consolidation tumor ratio of 0.25 or less [21]. In our research, the mean surgical margin in patients undergoing operation was strictly at least 19.5 mm, which was longer than Suzuki et al’ report (Table 4). Actually, no patient in the two groups suffered relapse at the surgical margin, which is different from Tsutani et al’ study. The reason may still be that our margins are longer. Wedge has probably been used for very peripheral lesion and segmentectomy is suitable for deeper lesion not accessible for a wedge. Even if it is difficult to examine in the study, segmentectomy should remain the standard to obtain large surgical margin. In our study, wedge resection actually had better margins. The reason may be that the surgeon may prefer to remove a larger portion of the lung during the operation of wedge resection.

Enough dissection of lymph nodes is significant not only to secure effect of radical excision but also to prevent tumor metastasis and recurrence. One study recommended that at least six dissection nodes to ensure proper TNM classification, but it was the requirement of lobectomy [22]. To those stage IA patients, it does not need so many [23, 24]. He et al. reported that sublobar resection patients with ≥3 evaluated lymph nodes are associated with better overall survival and lung cancer-specific survival [25]. Similarly, Dezube et al. defined lymph node sampling minimums in early stage NSCLC, retrospectively analyzing the National Cancer Database (NCDB) queried 2004–2014 for surgically treated clinical stage I/II NSCLC. These differences were not statistically significant until the number of 4 removed LN (respectively 3 for wedge-resections). For segmentectomies, median survival was not statistically associated with number of LN sampled. Based on NCDB data, LN sampling for lung cancer resections is recommended [26]. In our study, the median number of dissected lymph nodes during operation in simple segmentectomy was 4 and in the wedge resection group, there is only 1.5. The incidence of lymph nodes metastasis was 3 and 4%, respectively, which exist no difference (*p* = 0.08). The number of dissected lymph nodes doesn’t seem to be important for AIS or MIA. However, frozen section should be standard to rule out invasive adenocarcinoma, because complete lymph node dissection is still mandatory in this subgroup and wedge with one lymph node is not the appropriate treatment. There is a relevant issue, as second primary lung cancer is often seen in the follow-up of these patients. Baig et al. reported that anatomic resection has superior long-term survival compared with wedge resection for second primary lung cancer after prior lobectomy. Significant improvement in survival was observed with wedge resection for second primary lung cancer when adequate lymph node dissection was performed [27].

Another concern about postoperative pulmonary function changes in two groups. We could find postoperative pulmonary function in segmentectomy group recover more slowly than wedge resection group according to FVC, FEV1.0, DLCO% and PEF (Fig. 3). Isolating and splitting pulmonary segments, evening cutting into lung parenchyma, still had some effect on pulmonary function, especially the recovery of pulmonary function.

### Limitations

The present study had several limitations. First, it was a retrospective study and results are derived from 3 centers only in the Shanghai area. The limited data might have introduced bias. Second, because we use a propensity score matching method, the analyzed patients of segmentectomy group may be biased toward the similar characteristics of wide-wedge resection group after matching. Third, multiple regression analysis may have confounding bias.

## Conclusions

In summary, wedge resection may have comparable efficacy as simple segmentectomy for GGO diameter between 2 cm and 3 cm in NSCLC, but lead to less complications, less surgical procedure and faster recovery of pulmonary function. Moreover, securing operation margins and lymph nodes of dissection could be performed as effective measures whatever segmentectomy or wedge resection. Further investigations, larger samples and longer time following-up will be needed.

## Supplementary Information

**Additional file 1: Supplementary figure.** The 5-year RFS and OS in simple segmentectomy group was 93.1 and 91.9%, and in wedge resection group was 96 and 95.7% before propensity score matching.

**Additional file 2: Supplementary table**. All the patients and tumor characteristics of simple segmentectomy and wedge resection before propensity score matching.

## Data Availability

The datasets analysed during the current study are available from the corresponding author on reasonable request.
