# Supplementary figures and images for: Comparison of surgical outcomes and prognosis between wedge resection and simple Segmentectomy for GGO diameter between 2 cm and 3 cm in non-small cell lung cancer: a multicenter and propensity score matching analysis
Source: BMC Cancer. 2022 Jan 16;22:71. doi: 10.1186/s12885-021-09129-0 (PMC8761309; doi:10.1186/s12885-021-09129-0)

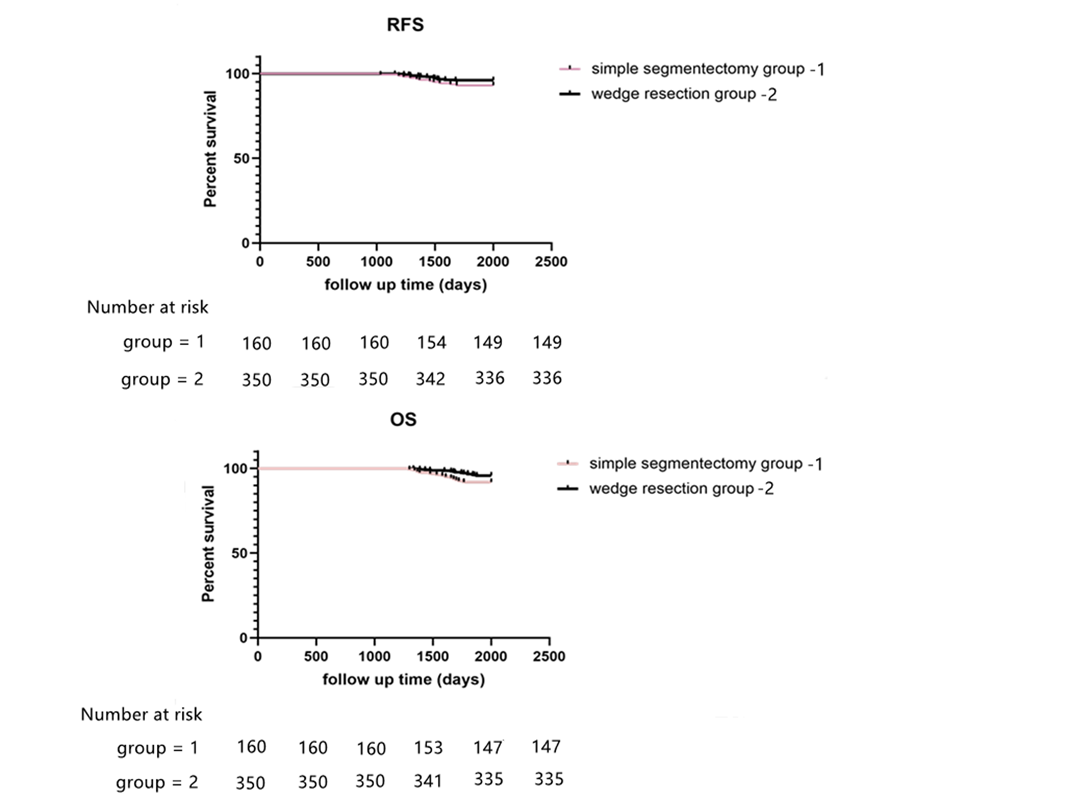

Supplement: Supplementary file 1 — Additional file 1: Supplementary figure. The 5-year RFS and OS in simple segmentectomy group was 93.1 and 91.9%, and in wedge resection group was 96 and 95.7% before propensity score matching. [file 12885_2021_9129_MOESM1_ESM.tif]
