# Supplementary material for: Comparison of surgical outcomes and prognosis between wedge resection and simple Segmentectomy for GGO diameter between 2 cm and 3 cm in non-small cell lung cancer: a multicenter and propensity score matching analysis
Source: BMC Cancer. 2022 Jan 16;22:71. doi: 10.1186/s12885-021-09129-0 (PMC8761309; doi:10.1186/s12885-021-09129-0)
Supplement: Supplementary file 2 — Additional file 2: Supplementary table. All the patients and tumor characteristics of simple segmentectomy and wedge resection before propensity score matching. [file 12885_2021_9129_MOESM2_ESM.docx]

**Supplementary table. All the patients and tumor characteristics of simple segmentectomy and wedge resection before propensity score matching**

| Variables | segmentectomy  n=160 | wedge resection  n=350 |
| --- | --- | --- |
| Comorbidities |  |  |
| Coronary artery disease | 17 | 41 |
| Diabetes mellitus | 25 | 57 |
| COPD | 31 | 50 |
| CTR | 0.86 | 0.57 |
| mGGO | 68 | 199 |
| pGGO | 92 | 151 |
| Clinical stage |  |  |
| cTisN0 | 43 | 104 |
| cT1miN0 | 60 | 117 |
| cT1aN0 | 16 | 31 |
| cT1bN0 | 23 | 35 |
| Pathologic stage |  |  |
| pTisN0 | 46 | 98 |
| pT1miN0 | 57 | 123 |
| pT1aN0 | 14 | 28 |
| PT1bN0 | 25 | 38 |
| Visceral pleural invasion | 4 | 6 |
| Lymphovascular invasion | 7 | 10 |
| Surgery |  |  |
| Right upper |  | 88 |
| S2 | 37 |  |
| Right middle | 14 | 36 |
| Right lower |  | 78 |
| S6 | 27 |  |
| S(7+8+9+10) | 8 |  |
| Left upper |  | 73 |
| S(1+2+3) | 25 |  |
| lingular segment | 18 |  |
| Left lower |  | 75 |
| S6 | 22 |  |
| S(7+8+9+10) | 9 |  |
| Postoperative data |  |  |
| stay in hospital (day) | 4.9 | 2.9 |
| [hospitalization](javascript:;) [expenses](javascript:;) ($) | 5212 | 3810 |
| drainage (day) | 3.1 | 2.1 |
| air leakage (>7 day) | 18 | 7 |
| Postoperative complications |  |  |
| overall | 32(20%) | 9(2.5%) |
| [pulmonary](javascript:;) [infection](javascript:;) | 10 | 3 |
| atelectasis | 11 | 5 |
| chylothorax | 8 | 0 |
| bleeding | 3 | 1 |
| Relapse patterns |  |  |
| local relapse | 0 | 0 |
| region relapse | 7 | 9 |
| distant relapse | 4 | 5 |
